# Supplementary material for: Biochemical analysis of nucleosome targeting by Tn5 transposase
Source: Open Biol. 2019 Aug 14;9(8):190116. doi: 10.1098/rsob.190116 (PMC6731594; doi:10.1098/rsob.190116)
Supplement: Supplementary Information [file rsob190116supp1.pdf]

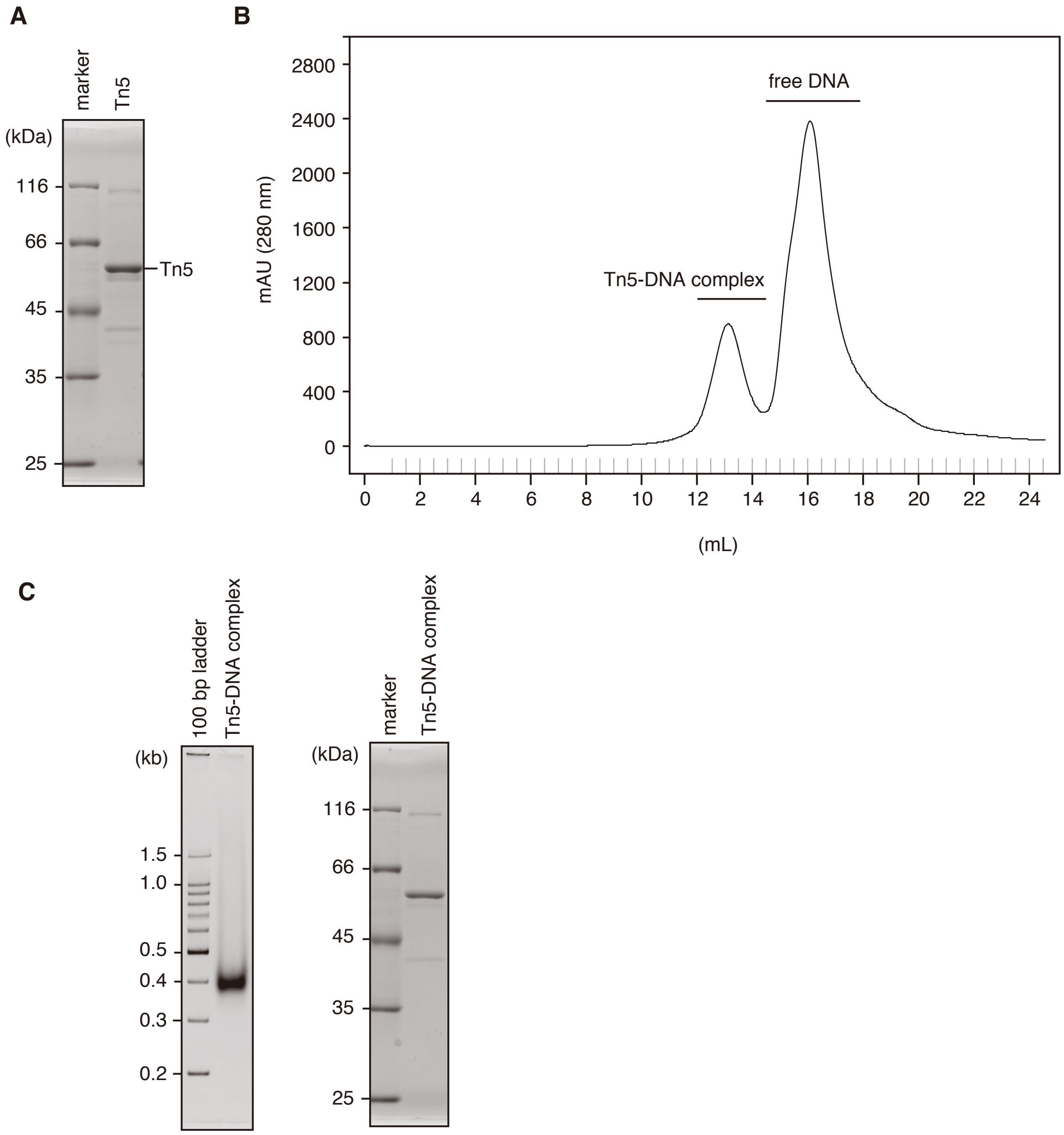

Supplementary figure S1. Preparation of the Tn5-DNA complex. (A) The purified Tn5 transposase was analyzed by denaturing polyacrylamide gel electrophoresis with Coomassie Brilliant Blue staining. (B) Representative results of size-exclusion chromatography to prepare the Tn5-DNA complex. Fractions containing the Tn5-DNA complex were collected. (C) The purified Tn5-DNA complex was analyzed by non-denaturing polyacrylamide gel electrophoresis with ethidium bromide staining (left panel), and denaturing polyacrylamide gel electrophoresis with Coomassie Brilliant Blue staining (right panel).

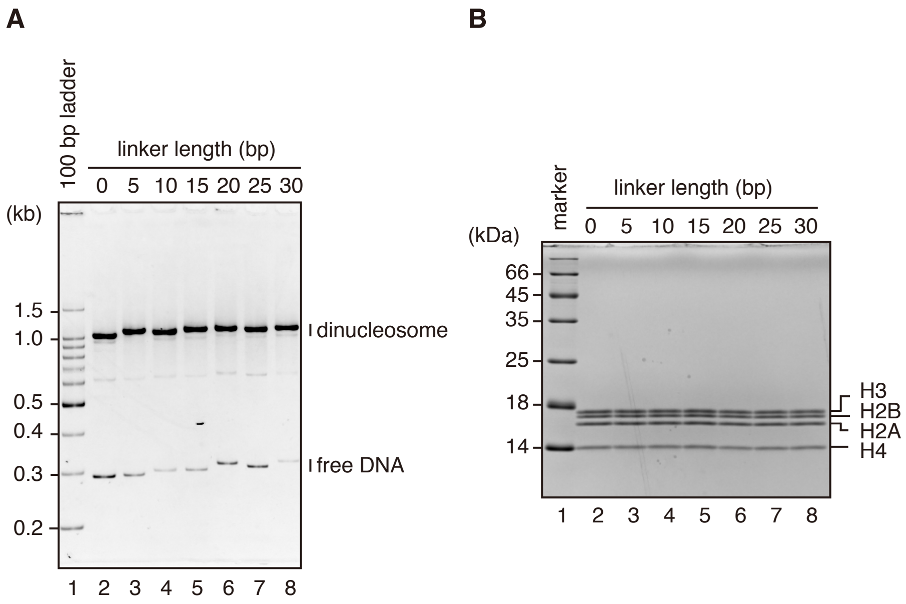

Supplementary figure S2. Preparation of dinucleosomes containing 0, 5, 10, 15, 20, 25, and 30 base-pair linker DNAs. (A) The reconstituted dinucleosomes were analyzed by non-denaturing polyacrylamide gel electrophoresis with ethidium bromide staining. The template-free DNAs exhibited unusual migration profiles, which do not correspond to the DNA length, probably due to the structural nature of the 601 sequence. The DNA sequences of these template DNAs were confirmed by direct sequencing. (B) The reconstituted dinucleosomes were analyzed by denaturing polyacrylamide gel electrophoresis with Coomassie Brilliant Blue staining.

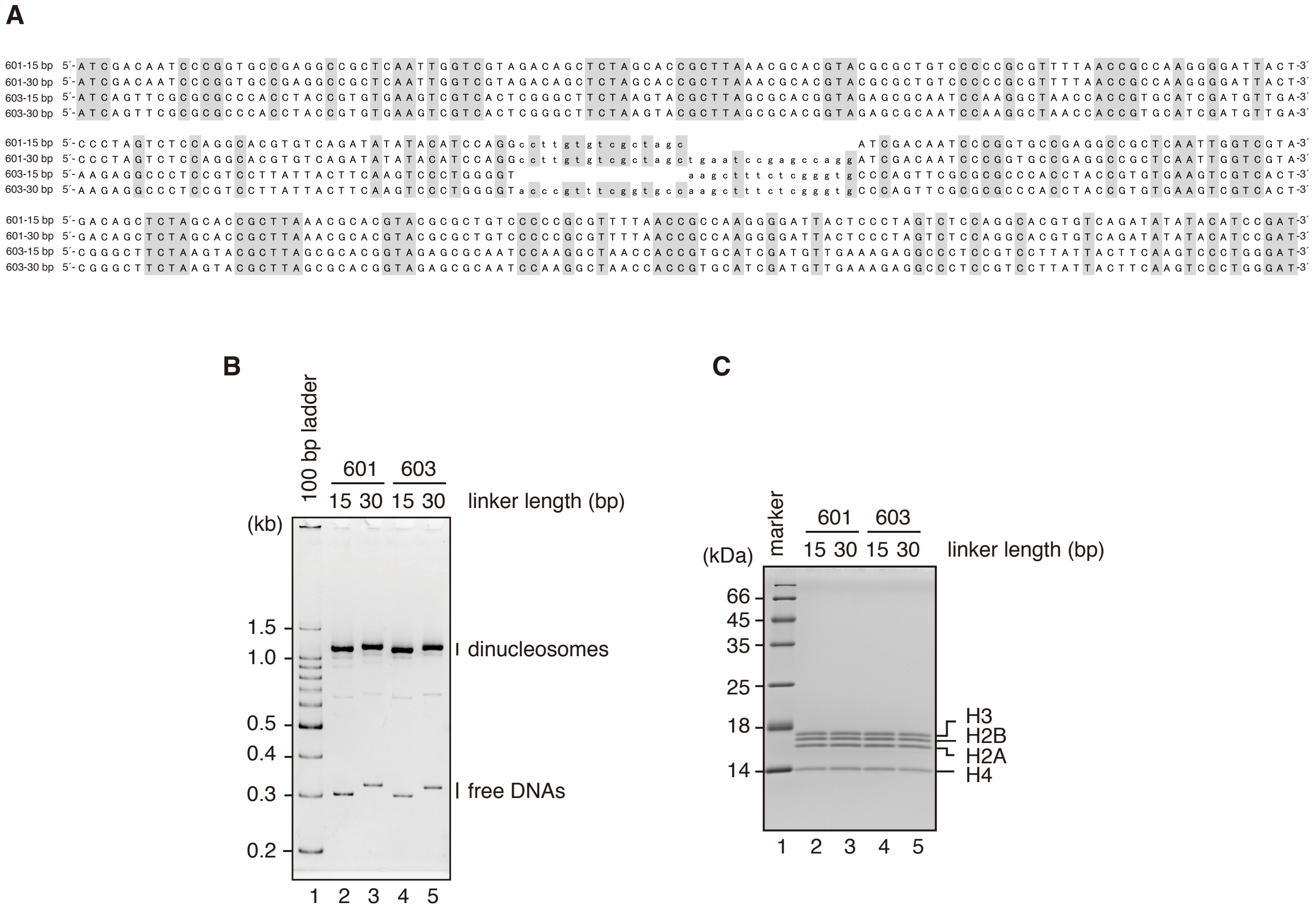

Supplementary figure S3. Preparation of dinucleosomes containing the 601 and 603 sequences. (A) DNA sequences of the substrates. Capital letters represent the 601 and 603 sequences. Small letters represent the linker DNAs. The reconstituted dinucleosomes were analyzed by non-denaturing polyacrylamide gel electrophoresis with ethidium bromide staining (B), and denaturing polyacrylamide gel electrophoresis with Coomassie Brilliant Blue staining (C).

601-0 bp-601

ATCGACAATCCCGGTGCCGAGGCCGCTCAATTGGTCGTAGACAGCTCTAGCACCGCTTAAACGCACGTACGCGCTGTCCCCCGCGTTTTAACC
GCCAAGGGGATTACTCCCTAGTCTCCAGGCACGTGTCAGATATATACATCCAGGATCGACAATCCCGGTGCCGAGGCCGCTCAATTGGTCGTAG
ACAGCTCTAGCACCGCTTAAACGCACGTACGCGCTGTCCCCCGCGTTTTAACCGCCAAGGGGATTACTCCCTAGTCTCCAGGCACGTGTCAGAT
ATATACATCCGAT

601-5 bp-601

ATCGACAATCCCGGTGCCGAGGCCGCTCAATTGGTCGTAGACAGCTCTAGCACCGCTTAAACGCACGTACGCGCTGTCCCCCGCGTTTTAACC
GCCAAGGGGATTACTCCCTAGTCTCCAGGCACGTGTCAGATATATACATCCAGGCCTTGATCGACAATCCCGGTGCCGAGGCCGCTCAATTGGT
CGTAGACAGCTCTAGCACCGCTTAAACGCACGTACGCGCTGTCCCCCGCGTTTTAACCGCCAAGGGGATTACTCCCTAGTCTCCAGGCACGTGT
CAGATATATACATCCGAT

601-10 bp-601

ATCGACAATCCCGGTGCCGAGGCCGCTCAATTGGTCGTAGACAGCTCTAGCACCGCTTAAACGCACGTACGCGCTGTCCCCCGCGTTTTAACC
GCCAAGGGGATTACTCCCTAGTCTCCAGGCACGTGTCAGATATATACATCCAGGCCTTGTGTCGATCGACAATCCCGGTGCCGAGGCCGCTCAA
TTGGTCGTAGACAGCTCTAGCACCGCTTAAACGCACGTACGCGCTGTCCCCCGCGTTTTAACCGCCAAGGGGATTACTCCCTAGTCTCCAGGCA
CGTGTCAGATATATACATCCGAT

601-15 bp-601

ATCGACAATCCCGGTGCCGAGGCCGCTCAATTGGTCGTAGACAGCTCTAGCACCGCTTAAACGCACGTACGCGCTGTCCCCCGCGTTTTAACC
GCCAAGGGGATTACTCCCTAGTCTCCAGGCACGTGTCAGATATATACATCCAGGCCTTGTGTCGCTAGCATCGACAATCCCGGTGCCGAGGCC
GCTCAATTGGTCGTAGACAGCTCTAGCACCGCTTAAACGCACGTACGCGCTGTCCCCCGCGTTTTAACCGCCAAGGGGATTACTCCCTAGTCTC
CAGGCACGTGTCAGATATATACATCCGAT

601-20 bp-601

ATCGACAATCCCGGTGCCGAGGCCGCTCAATTGGTCGTAGACAGCTCTAGCACCGCTTAAACGCACGTACGCGCTGTCCCCCGCGTTTTAACC
GCCAAGGGGATTACTCCCTAGTCTCCAGGCACGTGTCAGATATATACATCCAGGCCTTGTGTCGCTAGCTGAATATCGACAATCCCGGTGCCGA
GGCCGCTCAATTGGTCGTAGACAGCTCTAGCACCGCTTAAACGCACGTACGCGCTGTCCCCCGCGTTTTAACCGCCAAGGGGATTACTCCCTA
GTCTCCAGGCACGTGTCAGATATATACATCCGAT

601-25 bp-601

ATCGACAATCCCGGTGCCGAGGCCGCTCAATTGGTCGTAGACAGCTCTAGCACCGCTTAAACGCACGTACGCGCTGTCCCCCGCGTTTTAACC
GCCAAGGGGATTACTCCCTAGTCTCCAGGCACGTGTCAGATATATACATCCAGGCCTTGTGTCGCTAGCTGAATCCGAGATCGACAATCCCGGT
GCCGAGGCCGCTCAATTGGTCGTAGACAGCTCTAGCACCGCTTAAACGCACGTACGCGCTGTCCCCCGCGTTTTAACCGCCAAGGGGATTACT
CCCTAGTCTCCAGGCACGTGTCAGATATATACATCCGAT

601-30 bp-601

ATCGACAATCCCGGTGCCGAGGCCGCTCAATTGGTCGTAGACAGCTCTAGCACCGCTTAAACGCACGTACGCGCTGTCCCCCGCGTTTTAACC
GCCAAGGGGATTACTCCCTAGTCTCCAGGCACGTGTCAGATATATACATCCAGGCCTTGTGTCGCTAGCTGAATCCGAGCCAGGATCGACAATC
CCGGTGCCGAGGCCGCTCAATTGGTCGTAGACAGCTCTAGCACCGCTTAAACGCACGTACGCGCTGTCCCCCGCGTTTTAACCGCCAAGGGGA
TTACTCCCTAGTCTCCAGGCACGTGTCAGATATATACATCCGAT

603-15 bp-603

ATCAGTTCGCGCGCCCACCTACCGTGTGAAGTCGTCACTCGGGCTTCTAAGTACGCTTAGCGCACGGTAGAGCGCAATCCAAGGCTAACCACC
GTGCATCGATGTTGAAAGAGGCCCTCCGTCCTTATTACTTCAAGTCCCTGGGGTAAGCTTTCTCGGGTGCCCAGTTCGCGCGCCCACCTACCGT
GTGAAGTCGTCACTCGGGCTTCTAAGTACGCTTAGCGCACGGTAGAGCGCAATCCAAGGCTAACCACCGTGCATCGATGTTGAAAGAGGCCCT
CCGTCCTTATTACTTCAAGTCCCTGGGAT

603-30 bp-603

ATCAGTTCGCGCGCCCACCTACCGTGTGAAGTCGTCACTCGGGCTTCTAAGTACGCTTAGCGCACGGTAGAGCGCAATCCAAGGCTAACCACC
GTGCATCGATGTTGAAAGAGGCCCTCCGTCCTTATTACTTCAAGTCCCTGGGGTACCCGTTTCGGTGCCAAGCTTTCTCGGGTGCCCAGTTCGC
GCGCCCACCTACCGTGTGAAGTCGTCACTCGGGCTTCTAAGTACGCTTAGCGCACGGTAGAGCGCAATCCAAGGCTAACCACCGTGCATCGAT
GTTGAAAGAGGCCCTCCGTCCTTATTACTTCAAGTCCCTGGGAT

Supplementary figure S4. The sequences of the DNA fragments used for the dinucleosome reconstitution. The linker DNA regions are underlined.

Supplementary table S1. The major cleavage sites by the Tn5-DNA complex in the dinucleosome and naked DNA substrates.

sample	positions (counts)				
601-15 nucleosome upper strand	5´-141 (7,333)	5´-160 (1,921)	5´-140 (1,565)	5´-142 (1,155)	5´-161 (522)
601-15 nucleosome lower strand	5´-149 (10,721)	5´-148 (2,823)	5´-150 (2,492)	5´-168 (1,217)	5´-159 (207)
601-15 DNA upper strand	5´-207 (3,060)	5´-196 (2,529)	5´-217 (1,160)	5´-228 (922)	5´-206 (881)
601-15 DNA lower strand	5´-53 or 5´-215 (3,066)	5´-140 or 5´-302 (1,355)	5´-63 or 5´-225 (1,262)	5´-74 or 5´-236 (1,220)	5´-124 or 5´-286 (690)
601-30 nucleosome upper strand	5´-160 (4,605)	5´-141 (3,539)	5´-176 (1,353)	5´-175 (1,182)	5´-166 (738)
601-30 nucleosome lower strand	5´-149 (11,264)	5´-168 (2,901)	5´-148 (2,400)	5´-150 (2,254)	5´-157 (882)
601-30 DNA upper strand	5´-222 (5,119)	5´-211 (4,070)	5´-232 (2,099)	5´-221 (1,575)	5´-243 (1,506)
601-30 DNA lower strand	5´-140 or 5´-317 (1,783)	5´-53 or 5´-230 (1,682)	5´-74 or 5´-251 (1,336)	5´-79 or 5´-256 (936)	5´-63 or 5´-240 (896)
603-15 nucleosome upper strand	5´-161 (6,620)	5´-140 (2,415)	5´-160 (2,306)	5´-150 (2,302)	5´-143 (1,888)
603-15 Nucleosome lower strand	5´-169 (4,514)	5´-148 (4,341)	5´-158 (3,023)	5´-151 (2,608)	5´-160 (2,169)
603-15 DNA upper strand	5´-229 (170)	5´-176 (123)	5´-239 (108)	5´-178 (63)	5´-227 (63)
603-15 DNA lower strand	5´-85 (312)	5´-75 (222)	5´-92 (171)	5´-73 (74)	5´-90 (74)
603-30 nucleosome upper strand	5´-158 (5,814)	5´-176 (2,976)	5´-167 (1,968)	5´-165 (1,123)	5´-175 (947)
603-30 nucleosome lower strand	5´-146 (7,310)	5´-166 (5,829)	5´-175 (2,148)	5´-149 (1,966)	5´-151 (1,296)
603-30 DNA upper strand	5´-244 (3,339)	5´-254 (1,574)	5´242 (1,464)	5´-237 (1,450)	5´-191 (1,417)
603-30 DNA lower strand	5´-85 (3,793)	5´-75 (3,440)	5´-92 (1,908)	5´-73 (1,029)	5´-68 (943)
